# Pretreatment of Lignocellulosic Materials as Substrates for Fermentation Processes

**DOI:** 10.3390/molecules23112937

**Published:** 2018-11-10

**Authors:** Karolina Kucharska, Piotr Rybarczyk, Iwona Hołowacz, Rafał Łukajtis, Marta Glinka, Marian Kamiński

**Affiliations:** 1 Department of Process Engineering and Chemical Technology, Faculty of Chemistry, Gdańsk University of Technology, 80-233 Gdańsk, Poland; piotr.rybarczyk@pg.edu.pl (P.R.); iwona.holowacz@pg.edu.pl (I.H.); rafal.lukajtis@pg.edu.pl (R.L.); martglink@gmail.com (M.G.); markamin@pg.edu.pl (M.K.); 2 Department of Analytical Chemistry, Faculty of Chemistry, Gdańsk University of Technology, 80-233 Gdańsk, Poland

**Keywords:** lignocellulosic biomass, biohydrogen, pretreatment, hydrolysis, dark fermentation

## Abstract

Lignocellulosic biomass is an abundant and renewable resource that potentially contains large amounts of energy. It is an interesting alternative for fossil fuels, allowing the production of biofuels and other organic compounds. In this paper, a review devoted to the processing of lignocellulosic materials as substrates for fermentation processes is presented. The review focuses on physical, chemical, physicochemical, enzymatic, and microbiologic methods of biomass pretreatment. In addition to the evaluation of the mentioned methods, the aim of the paper is to understand the possibilities of the biomass pretreatment and their influence on the efficiency of biofuels and organic compounds production. The effects of different pretreatment methods on the lignocellulosic biomass structure are described along with a discussion of the benefits and drawbacks of each method, including the potential generation of inhibitory compounds for enzymatic hydrolysis, the effect on cellulose digestibility, the generation of compounds that are toxic for the environment, and energy and economic demand. The results of the investigations imply that only the stepwise pretreatment procedure may ensure effective fermentation of the lignocellulosic biomass. Pretreatment step is still a challenge for obtaining cost-effective and competitive technology for large-scale conversion of lignocellulosic biomass into fermentable sugars with low inhibitory concentration.

## 1. Introduction

Lignocellulose is a basic component of plants and is widely exploited by various branches of industry, e.g., pharmaceutical, food and cosmetic industries. Lignocellulose is also a substrate for the production of fillers for thermoplastic composite materials [1], bioethanol [2], beauty masks, curative chows, and many other products. Some lignocellulosic materials include grasses, trees, stems, and flowers as well as fast-growing energetic plants, e.g., energetic willow, poplar and Miscanthus. Therefore, lignocellulosic biomass is one of the important groups among renewable resources. Residues from sawmills, the forest and paper industry, waste paper and wastes coming from agriculture, i.e., cereal straw, corncob and corn straw, potato haulms, parts of sugar beets and the residue from sunflowers and rapeseed oil pressing, are the main sources of lignocellulosic materials [3,4,5].

In the current paper, the term lignocellulosic biomass includes different kinds of wastes that exit as small pieces and particles from the abovementioned sources and any other waste fragments of plants and firewood. The physical form and size of the lignocellulosic material structure determines the pretreatment methods that should potentially be used, including any necessary high energy-need mechanical pretreatment.

Waste lignocellulose originating from both food and non-food industries are believed to be a promising material for the production of second generation biofuels, including biohydrogen [6,7,8,9,10], bioethanol [11,12,13], biogas [13,14,15], synthetic biofuels [16,17], methane [18,19,20], biodiesel [21,22], etc. The hydrogen energetic value, which is the amount of energy generated from 1 kg of material, is the highest among all known fuels, except ones produced from nuclear decay (Table 1). At the same time, hydrogen, as a fuel, is environmentally friendly, as no greenhouse gases (i.e., carbon dioxide) are emitted during its combustion [8,23,24].

Hydrogen is an elementary substrate in various chemical and petrochemical processes, i.e., synthesis of aniline from nitrobenzene, production of synthetic gas, olefins hydrogenation, synthesis of ammonia, methane, methanol, production of plastics, integrated circuits and optical fibers [25]. Hydrogen is also used in the petrochemical processes of hydrorafination, hydrocracking and hydroconversion. The energetic potential of hydrogen is greater than the potential of any other chemical or biological energy source, as shown in Table 1, unless nuclear decay is considered.

Hydrogen is produced mainly via the following processes: steam methane reforming, water gas shift, coal and coke gasification and water gas reforming of hydrocarbons originated from crude oil [8]. Water electrolysis is another method of hydrogen generation [8]. However, it requires a high input of electric energy. It is believed that in the near future, biomass and solar pathways for hydrogen production will need to be initiated at a larger scale [32]. Therefore, it is important to search for bio-derived liquids and microbial biomass conversion methods that can be used at the industrial scale.

An increase in fossil fuels exploitation and their depletion together with the negative environmental impacts resulting from their combustion, i.e., greenhouse effect, air pollution and smog formation, have resulted in changes to governmental energetic policies, especially in Europe, and to a dynamic development of investigations concerning renewable energy resources [33]. A main target of the climatic package implemented by the European Union (EU) is to reduce the emissions of greenhouse gases by means of highly efficient and innovative technologies [34]. In the EU, approximately 67% of the primary energy obtained from renewable resources comes from biomass. The calorific value of the biomass is approximately 14–19 MJ/kg. Furthermore, a total of 370 TWh of bioenergy electricity was produced in 2012. This corresponds to 1.5% of the world electricity generation, according to International Energy Academy. Numerous technologies for generating bioenergy heat and power already exist, ranging from solid wood heating installations for buildings to biogas digesters for power generation and large-scale biomass gasification plants. Co-firing biomass and coal in existing power plants can be an important option to achieve short-term emission reductions and make the use of existing assets more sustainable. In addition, new dedicated bioenergy plants are becoming important to meet the growing demand for bioenergy electricity and heat.

Approximately 48% of the biomass-originating energy comes from the processing of lignocellulosic materials [33,34]. However, the development of technologies for lignocellulosic biomass processing focuses mainly on biorefining. The main products of biorefining are biofuels, biomaterials and biochemicals, among which, bioethanol and furan biofuels are mentioned as the most important [32,33,35]. In favorable circumstances, producing energy from biomass can be cost competitive today, in particular, concerning heat generation. Such support is justified by the environmental, energy security and socioeconomic advantages associated with sustainable bioenergy, but should be introduced as a transitional leading agent to cost competitiveness in the medium term. The means and measures should be supported by a strong policy framework that balances the need for energy with other important objectives, such as greenhouse gas reduction, food security, biodiversity, and socioeconomic development [32]. According to IEA the energy scenario changes every year. The energy power generation forecast shows that the amount of energy produced in the world raised from 200 TWh (2006) to 350 TWh (2006) and 380 TWh (2016) and is expected reach 600 TWh in 2020 [36].

The biggest limitation regarding exploitation of lignocellulose as a substrate for biohydrogen production via fermentation methods is the problem of efficient lignocellulose hydrolysis to sugars during hydrogen fermentation. Thus, a proper lignocellulose pretreatment is an indispensable step in biohydrogen production. The main aims of the pretreatment include the disintegration of a tight structure of lignocellulosic complexes and an increase in the accessibility of various hydrolytic factors towards the cellulose. During hydrolysis, as a result of chemical and biochemical processes catalyzed by enzymes, together with physical and physicochemical treatment, a decomposition of organic matter occurs. The resulting simple chemical compounds are metabolized by microorganisms during the fermentation processes. The increase of biohydrogen production efficiency requires the development of economically and environmentally friendly technologies for lignocellulosic biomass pretreatment [37].

## 2. Composition and Structure of Lignocellulosic Biomass 

Lignocellulose is composed of three main fractions: cellulose (30–60% of dry matter), hemicellulose (14–40% of dry matter) and lignin (7–25% of dry matter) [34]. These polymers are associated with each other in a hetero-matrix, and the relative composition depends on the type, species, and even the source of the biomass, which varies as shown in Table 2. The relative abundance of cellulose, hemicellulose, and lignin are the key factors for potential energy productivity. Lignocellulose is a widely available material [38] that cannot be consumed as food by people or animals. Table 2 presents the shares of the main fractions in selected examples of lignocellulosic biomass. Composition values for average material of high importance in worldwide agriculture have been presented to highlight the diversity of their content.

Composition values of main components concerning cellulose, hemicellulose and lignin in biomass samples may vary to a wider limit. For example, content of cellulose in bast fibers is reported at level between 60–70%, in paper 85–99%, in waste industrial paper 70–80% whereas in residues of textile industry and cotton processing (lint, fluff) up to 90%. Content of lignin in various biomasses can vary from 5% (in bast fibers and waste paper/cardboard) to 48% in olive husk [42,43,44,45,46].

Cellulose is an unbranched crystalline structured biopolymer composing the cell walls of plants as well as bacteria, fungi, and algae. Cellulose is composed of several to hundreds of thousands of units of glucose, connected by β-1,4-glicosidic bonds. Enzymatic hydrolysis of cellulose may be realized with the application of endoglucanase (hydrolyzes internal β-1,4-glicosidic bonds), exoglucanase (removes mono- and dimers from the end of the glucose chain) and glycosidase (hydrolyzes glucose dimers). Enzymatic hydrolysis is assisted by solutions of diluted and concentrated acids. The degradation of cellulose is difficult due to the high stability of cellulose microfibers and the polysaccharidic coating surrounding them [47].

Hemicellulose is a branched heteropolymer composed of hexoses (d-galactose, l-galactose, d-mannose, l-fructose), pentoses (l-rhamnose, arabinose, xylose), d-glucuronic acid and acetylated sugars. The proportions of sugars composing hemicellulose differ depending on the plant type, cultivation place and season. Chemical hydrolysis of hemicellulose occurs easier in comparison with cellulose. The qualitative composition of hemicellulose depends on the material of origin. For example, hemicellulose derived from straw or grass is composed mainly of xylane. Hemicellulose derived from coniferous trees or from the maidenhair tree is composed mainly of glucomannan [5]. Glucomannan may only be extracted in alkaline conditions, while xylane starts to dissolve at a pH of approximately 10 [48]. Therefore, phanerogams are rarely used as a feed for dark fermentation processes [47]. It is estimated that the elimination of approximately 50% of hemicelluloses from the processed raw material may considerably increase the yield of cellulose degradation but also decreases the formation of bio toxic compounds, such as furfural and its derivatives [49]. Hemicellulose is the most susceptible to the thermal and chemical processing fraction of lignocellulose [37].

Lignin is an amorphous, water-insoluble heteropolymer. Lignin is a product of condensation of three monomeric phenol alcohols: trans-*p*-cumarylic, trans-*p*-coniferylic, trans-*p*-sinapylic. Lignin is a component of a cell wall and its main biological function is to form an impermeable structure that protects a plant from an invasion of microbes [37,50]. The presence of lignin and cellulose-lignin structures in biomass are responsible for its ineffective hydrolysis and fermentation, because both mentioned fractions are water-insoluble. The elimination of lignin results in an increase in the biomass digestibility [51]. The presence of lignin inhibits the biomass hydrolysis due to the toxicity of lignin derivatives as well as non-specific adsorption of hydrolytic enzymes within the structure of lignocelluloses. The delignification, i.e., the extraction of lignin by means of chemicals, leads to biomass swelling. The biomass swelling is an alteration of lignin structure, which results in an increase of the area of lignocellulose fibers exposed to the cellulolytic enzymes [52].

### Different Approaches Towards Lignocellulosic Biomass Pretreatment

Lignocellulosic materials possess a great potential to be a good feedstock to produce biofuels as well as valuable chemicals. Pretreatment is an indispensable step of lignocellulosic biomass processing due to the complex structure and recalcitrant nature of such a feedstock [53,54]. When the production of biofuels is considered, the pretreatment is focused on improving the cellulose and hemicellulose accessibility resulting in increased efficiency of further processing stages i.e., saccharification. As a result, of the pretreatment, complex lignocellulosic structures are converted to simple components (cellulose, hemicelluloses, and lignin) which is generally reflected by the removal of lignin, preservation of hemicelluloses, reduction of cellulose crystallinity and an increase of the material porosity. The pretreatment is regarded as efficient when the formation of sugars in further enzymatic step of biomass processing is enhanced as well as the degradation of carbohydrates and the formation of inhibitory compounds are limited to minimum [55,56,57].

On the other hand, biomass pretreatment may be focused on the fractionation of biomass components towards valuable chemical compounds obtained via environment-friendly routes of biomass processing [58]. For example, the production of ethyl levulinate, an additive in gasoline and diesel, may be obtained via ethanol-aided acid-catalyzed method that follows the cost-effective mechanical deconstruction of biomass into ultrafine particles and mild processing temperatures (~160 °C) [53]. Additionally, separation of compounds (e.g., formed during the pretreatment i.e., inhibitors for further processing of biomass (enzymatic hydrolysis, fermentation) may aid the processing efficiency as well as enable the production of valuable chemical compounds [59].

Three main fractions of lignocellulosic materials i.e., cellulose, hemicelluloses, and lignin, represent potential feedstocks for bio-sourced chemicals. However, due to their varied chemical functionalities, separation steps are indispensable to isolate the appropriate fraction and break it into its individual building units [60]. 

The joined strategy of biofuels production together with energy generation and production of valuable chemicals from lignocellulosic materials is defined as a bio refinery concept. In such an approach, the pretreatment operations, being cost-intensive, are focused not only at enhancing the biofuels production, but also on the production and further separation of useful chemical compounds [56]. The fractionation of biomass into its individual building blocks remain a bottleneck and a major challenge to the bio refinery concept [60].

The approaches towards the methods and effects of biomass pretreatment have been evolving from conventional, cost-intensive methods employing harsh chemicals and conventional heating towards more environmentally friendly (biological methods) and more efficient methods (green chemicals, large-scale sustainable technologies). Such a shift is of special importance because of complex structure and recalcitrance of lignocellulosic biomass, pretreatment steps are crucial for the effective biomass conversion. Thus, the development of efficient technologies is still a challenge in the field [61]. The research into novel methods of biomass pretreatment, including e.g., non-ionizing and ionizing radiation, pulsed-electric field, high pressure, ultrasounds, supercritical solvents, organosolv or application of deep eutectic solvents is additionally justified because the pretreatment step accounts for up to 40% of total costs of biomass processing towards biofuels or value-added products [56,62,63,64,65].

Lignocellulose, being the most abundant, cheapest, and easiest grown form of biomass, is an interesting feedstock for the production of biofuels and valuable chemical compounds. The environmental and economic sustainability of the biomass processing, including especially the pretreatment steps, remains as a research challenge. It is proposed that high-value by-products and gas-liquid-solid pyrolysis products should be improved in terms of pretreatment processes instead of the chemical reaction rates. Additionally, novel industrial-scale approaches for biomass processing towards both biofuels and valuable chemical production seems to be relevant for the management of inhibitory compounds formed during the pretreatment [54,66].

## 3. Methods of Lignocellulosic Biomass Pretreatment

Biomass recalcitrance containing mainly cellulose and lignin for fermentation is related to the content of lignin and its subunits, called syringol and guaiacol. The presence of the abovementioned chemical compounds causes problems with the chemical as well as biochemical degradation of the biomass [47]. According to Hendriks and Zeeman, hemicellulose is the least resistant to the thermal and chemical processing component of lignocellulose [37]. Currently, the preparation procedures for the optimal formation of lignocellulosic biomass and its pretreatment prior to fermentation are widely investigated [3,4,5,9,34,39,47,48,49,50,52,67].

An initial pretreatment of lignocellulosic biomass is necessary for the efficient progress of various processes, enabling the extraction of valuable products from the biomass, i.e., during the fermentation processes. The pretreatment of lignocellulosic materials includes fragmentation of solids, alteration of the lignocellulose structure, increasing the contact area between the material and the chemical or enzymatic reagents and the reduction of the degrees of crystallization and polymerization of the cellulose [68]. The pretreatment methods should be economically viable and should not lead to the formation of toxic substances or chemical compounds inhibiting the enzymatic and fermentation processes [34]. 

The physical, chemical, physicochemical, and biological methods of lignocellulosic biomass pretreatment are mentioned and described as follows [34]. The main aim of physical pretreatment is the reduction of the size of the biopolymer particles by means of fragmentation, grinding, milling, hacking, rolling and mechanical interactions [69]. Physical pretreatment also includes such methods as microwave radiation, sonication, spray drying, gamma radiation and pyrolysis. Table 3 presents some selected pretreatment methods with their basic characteristics.

Chemical methods enable the decomposition of lignocellulose to simple compounds because of chemical reactions in aqueous solutions—acid and alkaline pretreatment, oxidation, ozonolysis as well as dissolution in ionic liquids or organic solvents [3,6,34,70,71,72,73,74,75,76,77,78,79,80]. 

Physicochemical methods enable the decomposition of the lignocellulose structure by means of oxidation combined with thermal treatment. The methods include steam explosion, carbon dioxide explosion and ammonia fiber explosion (AFEX) [78,79,80].

Biological methods aim at the treatment of hemicellulose and lignin by means of fungi, bacteria, and enzymes decay. *Auricularia auricula* and *Trichoderma reesei* are widely applied [17,29,79].

The methods of lignocellulosic biomass pretreatment mentioned above are presented in Table 3 and further discussed in Section 3.1, Section 3.2, Section 3.3, Section 3.4 and Section 3.5

### 3.1. Physical Methods of Lignocellulosic Biomass Pretreatment

The application of physical pretreatment positively effects the hydrolysis efficiency and the anaerobic decomposition of the plants’ biomass to liquid and gaseous fuels and other valuable organic products [81]. According to Dong-Hoon et al., biohydrogen production during fermentation increases when mechanical/physical pretreatment is applied [43]. It was shown that mechanical pretreatment may increase the biomass temperature to approximately 70 °C. It was also shown that the delignification and depolymerization taking place during the biomass pretreatment may considerably limit the amounts of side-products (furan and its derivatives) generated during hydrolysis and fermentation [43]. 

On the other hand, Hendriks and Zeeman doubt the economic viability of fermentation processes that are preceded by mechanical pretreatment. This is caused by a low increase in biohydrogen production correlated with the high energy-need operations of the mechanical fragmentation of biomass [37].

In most papers cited in this work, biomass fragmentation is applied as a pretreatment step. However, the availability of information concerning process parameters and energy balances is low. It is generally stated that the concentrations of biomethane, bioethanol and biohydrogen are approximately 5–25% greater for fragmented biomass compared to non-fragmented structures [37]. In the case of several investigations, chemical pretreatment was also applied prior to mechanical or physical pretreatment [23,49]. It is especially beneficial towards so called soft agricultural wastes, i.e., waste from potato and corn cultivation. Pretreatment of biomass of the mentioned type requires lower energy input than that for conifers. When soft wastes are treated with 5% sodium hydroxide, the grinding time is reduced to half as the biomass becomes more fragile and brittle [23,49].

Soft biomass mechanical shredding performed at an increased temperature (50–70 °C) leads to rupturing of lignocellulose fibers and reduces the time needed for material digestion during further treatment by approximately 23–59% [75]. When the shredded material is fractionated, i.e., by a sieves system, the efficiency of the hydrolysis is higher, the smaller the particles are used [75]. Reducing the size of the feed biomass increases the material’s specific area and decreases the degree of polymerization and crystallization of the lignocellulose [34]. However, mechanical pretreatment is energy-intensive and, thus, its application must be related with a reasonable increase of final energy output. 

Mechanical size reduction using a high-pressure fluidizer can enable the complete deconstruction of the cell wall to nanofibers in woody biomass. The results indicate that the lignocellulose nanofiber is almost completely hydrolyzed by the enzymes within a short time [142]. A relatively high energy input is required to achieve a high enzymatic hydrolysis rate and fermentable sugar yield. However, it provides a nonchemical, green route for the pretreatment of lignocelluloses, without the production of undesirable compounds, and facilitates the downstream conversion and processing [142].

Radiation energy of 300–700 W/m^3^ is usually applied when microwaves are used for lignocellulose biomass pretreatment. As a result of the microwave action, intermolecular water is evaporated, and hydrogen bonds are broken. Thus, the stability of the lignocellulose molecules decreases. On the other hand, cellulose is rather insensitive to microwave radiation when compared to water molecules. At the same time, the decrease in the size of the cellulose crystals would considerably increase the efficiency of the biomass hydrolysis [6]. Application of microwave radiation prior to the chemical hydrolysis of the lignocellulosic material enables the cellulose content to reach approximately 56% in the hydrolysate [3]. The content of reducing sugars in hydrolysates may be increased four times when microwaves (400 W, radiation time 20 min) are used together with either acid or alkaline pretreatment (1%, 2% or 3% sulfuric acid or sodium hydroxide) [117,143].

The new alternative to deconstruction of the lignocellulose structure is a pretreatment using microwaves and glycerol. Microwaves applied during the pretreatment for lignocellulosic biomass suspended in a solution of alkaline results in an increase in the degree of delignification by 12–30% for corn and rice wastes [143]. The results of microwave irradiation of sugarcane bagasse for 5 min with distilled water, phosphoric acid (pH 3.0) and glycerol (10%) indicate, that 5.4 and 11.3% *w*/*w* of lignin and xylan fractions are degraded, respectively. Sugarcane bagasse was subjected to microwaves. Furthermore, the highest yields from the enzymatic hydrolysis of hemicellulose (22.4%) and cellulose (40.2%) *w*/*w* were obtained for bagasse treated with microwave irradiation and glycerol after 24 h of incubation [144].

The application of ultrasounds as a biomass pretreatment method results in a decreased fermentation time because of the disintegration of the lignocellulosic structures [35]. The efficiency of the pretreatment depends on the selection of the oscillation frequency, process temperature and time as well as the type of treated material [8,84,85]. Usually, a sonication frequency of 20–40 kHz is applied, and the biopolymer’s internal hydrogen bonds are broken. The biomass undergoes the loosening, swelling, and rupturing of fibers [87]. The best results of delignification were noted for a sonication frequency of 40 kHz, with respect to fragmented biomass [87]. 

Another method of biomass pretreatment is the application of electron beams or gamma radiation. Gamma radiation and electron beams cause the rupture of 1,4-glycosidic bonds and leads to an increase in the specific area of the lignocellulosic biomass as well as a decrease in its crystallinity degree. The electron beam is low penetrating and the entire energy of electrons is deposited in relatively thin layers of material. Gamma radiation is highly penetrating [89]. Therefore, it may be applied to thick materials. Pretreatment using gamma radiation results in the degradation of biomass, overcoming the biomass recalcitrance, as well as increasing its solubility and decreasing the mechanical strength. The effects mentioned above depend on the composition and the degree of biomass fragmentation [47,49]. However, even though the cellulose crystallinity decreases from 51% to 32%, gamma radiation is not an industrial pretreatment method, mainly due to high costs and related environmental and safety reasons [47,89,90]. The use of electron beam processing (EBP) for pretreatment of sugarcane bagasse increased the conversion yield of cellulose to glucose from 8%–12% after 24 h of incubation and to 15% after 48 h [145]. EBP followed by thermal treatment (60 min at 180 °C) increased the yield of the enzymatic hydrolysis of cellulose, reaching 71.55%. Hemicelluloses were totally hydrolyzed after EBP [145]. 

Pyrolysis is an alternative biomass pretreatment method [50]. It consists of the thermochemical decomposition of biomass. The decomposition starts at a temperature of approximately 200 °C. Intensive gasification together with cellulose carbonation occurs at temperatures above 300 °C. As a result, of pyrolysis, carbon, and pyrolytic gases, including hydrogen and carbon monoxide, are formed. Pyrolysis is not widely applied for hydrogen production; however, it is used to obtain syngas and bio-oil. 

### 3.2. Chemical Methods of Lignocellulosic Biomass Pretreatment

The aim of mechanical and physicochemical operations is to initially prepare the biomass material for further treatment. The next stages of lignocellulosic biomass processing are chemical or enzymatic treatments. The most popular chemical method is alkaline or acid hydrolysis. Moreover, oxidation agents, ionic liquids and organic solvents are applied prior to hydrolysis. Table 4 presents exemplary conditions of chemical hydrolysis and the way they correspond with efficiencies of biomass conversion, estimated as the concentrations of monosugars after the hydrolysis. 

According to Table 4, either aqueous solutions of sodium or calcium hydroxides (alkaline pretreatment) or sulfuric acid (acid hydrolysis) are used for the chemical pretreatment. Application of the chemical pretreatment resulted in an increase of monosugars concentration compared to the initial concentration in the biomass. The efficiency of the alkaline pretreatment is higher than that of the acid hydrolysis for processes realized at the same temperature and hydrolysis time [51]. A discussion regarding the chemical methods of biomass pretreatment is given in further sections of this work. 

#### 3.2.1. Acid Pretreatment of Lignocellulosic Biomass

Acid pretreatment of lignocellulosic biomass aims at increasing the sugar substrate digestibility, defined as the concentration of reducing sugars after the hydrolysis, by microorganisms [79]. Sulfuric or hydrochloric acids are usually used for hydrolysis in concentrations varied between 0.5–10% *v*/*v*. When the biomass is in the form of straw or small branches, no fragmentation of material is required prior to acid hydrolysis [53].

Acid hydrolysis is realized under increased pressure (above 2 Ba) and temperature. Acid hydrolysis may be realized as a continuous process at temperatures above 160 °C (acid concentrations from 5–10%) or as a periodic process at temperatures below 160 °C (acid concentrations of 10–40%) [35,37,93]. 

Acid hydrolysis consists of damaging the lignin structure, dissolution of the hemicellulose and aiding the decomposition of cellulose to simple sugars. Efficient acid hydrolysis must be realized at an increased temperature. However, temperatures above 110 °C cause toxic compounds, such as furfural and 5-hydroxymethyl furfural, to form. The mentioned substances inhibit enzymatic and microbial hydrolysis and thus must be eliminated. Elimination of inhibitors may be realized by adsorption on activated carbon or via precipitation with calcium hydroxide [37,92]. Due to the increased temperature, other inhibitors such as chloric, phosphoric, or nitrous acids are formed, depending on the hydrolyzing agent. After the adaptation process, specific methane bacteria may tolerate such inhibitors in their environment, but only when very low concentrations occur.

Acid hydrolysis is an attractive pretreatment method as the hemicellulose degradation runs with the efficiency of approximately 20–90%, depending on the process conditions (Table 4) [35,37,39,93]. 

It is known that the concentration of acid during hydrolysis, independent from the temperature, varies from 10–30% *v*/*v* [35,37,91,92,93,94]. However, investigations regarding acid concentrations revealed that a concentration of approximately 3% is enough to obtain high hydrolysis efficiency [92]. A low acid concentration requires the temperature to be increased to approximately 60 °C to obtain an increased rate of hemicellulose saccharification [91]. However, the inhibitors concentration in hydrolysates is lower when a high concentration of acids is used. 

Alternatively, the biomass may be treated with highly concentrated sulfuric acid (above 30%) at ambient temperature. This approach results in highly efficient hydrolysis and a relatively high concentration of released sugars (up to 70% of glucose). However, high concentrations of acid cause the corrosion of equipment [34,95]. After the completion of the acid hydrolysis, the pH must be regulated to the values required for enzymatic or microbial treatment [34,37,94,95].

Regardless of the processed lignocellulose material, the approach to its hydrolysis may be two staged [34,37]. It decreases the possibility of sugar degradation after hydrolysis. During two-stage hydrolysis, first, hemicellulose is dissolved in mild conditions (140–190 °C, pressure above 4 Ba), then cellulose hydrolysis occurs (190–230 °C, pressure above 6 Ba). Kootstra et al. investigated the hydrolysis of wheat straw using sulfuric, fumaric and maleic acids at a concentration equal to 10% [96]. The processes were performed at temperatures of 130, 150 and 170 °C for 30 min and under a maximum pressure of 200 Ba. At the highest of the investigated temperature values, the efficiency of glucose production reached 98% using sulfuric acid and 96% using maleic acid. The process efficiency with fumaric acid was distinctly lower (efficiency below 20%). The highest efficiency of xylose formation (approximately 80%) was obtained using H_2_SO_4_ at 150 °C (pressure below 200 Ba). Xylose efficiency decreased when the temperature exceeded 170 °C due to the degradation of xylose to furfural. The maximum efficiency of xylose was obtained using maleic acid at 170 °C (pressure below 200 Ba). 

Nitric and phosphoric acids are also used for the hydrolysis of biomass coming from leguminous plants, grasses, corn as well as birch, poplar, maple, and aspen wood [91]. It was observed that the crystallinity index is not a function of the pretreatment temperature, but its increase is related to the elimination of amorphous cellulose structures during hydrolysis. The crystallinity index is defined as the degree of substance crystallinity. 

Liu et al. investigated the pretreatment of corn straw using sulfuric acid at concentrations equal to 2%, 4% and 6% and temperatures of 80, 100 and 120 °C [84]. It was found that using 2% sulfuric acid at 120 °C (reaction time 43 min) resulted in the efficiency of xylose formation equal to 77%, while glucose efficiency was only 8.4%. It is suggested that the hydrolysis time, as well as the process temperature and acid concentrations, must be selected depending on the biomass type [84].

Hernandez et al. used dilute sulfuric acid (1 g per 100 g of the reaction mixture) pre-hydrolysis as the pretreatment for the enzymatic hydrolysis of cellulose from residual empty pods of *Moringa oleifera* fruits. The cellulose recovery in the pretreated solids obtained under a temperature 130 °C and a reaction time 10 min was above 90%. Under a temperature of 190 °C and a reaction time 30 min, the cellulose recovery decreased to 81–85%, which can be explained by the partial solubilization of the easily hydrolysable fraction. The highest sugar concentration in the acid hydrolysates and the highest conversion (84.3%) of cellulose in the enzymatic hydrolysis were obtained during the pretreatments performed at 160 °C for 20 min. The furfural concentration reached 4.04 g/L at 160 °C for 20 min and decreased with the increase in the temperature and reaction time. Hydroxymethylfurfural (HMF), formic and levulinic acids were formed only during the pretreatments under the highest temperature [151].

Kumar et al. used an alkaline treatment for delignification of biomass before the two-stage acid hydrolysis. The study used sodium hydroxide concentrations of 0.5%, 1%, 2%, 3% (*w*/*v*), and the process was conducted at room temperature for 6, 12, 18 and 24 h. The highest rate of removal of lignin (52.48%) was obtained with a 3% concentration of the catalyst after 24 h [3].

#### 3.2.2. Alkaline Pretreatment of Lignocellulose Biomass

Alkaline pretreatment consists of the solvation of lignocellulose particles and hydrolytic decomposition of lignocellulose, called saponification. The biomass undergoes swelling, which aids the processes of hydrolysis and fermentation [3]. As a result of the alkaline pretreatment, the crystallinity index decreases, and the specific area of the biomass increases. Consequently, the lignin structure is altered and undergoes unfolding [71,99,100]. Some popular alkaline reagents used as pre-treating agents are sodium or calcium hydroxides as well as ammonia. Application of the mentioned reagents enables the degradation and dissolution of lignin and exposes the cellulose to the hydrolyzing agents [101,102,152]. Lignin is dissolved and undergoes recombination. As a result, of recombination, the lignin structure is changed, similarly to the changes caused by mechanical treatment. Importantly, the energy input is much lower for the alkaline pretreatment in comparison with mechanical pretreatment. Furthermore, in alkaline conditions, xylan may be extracted from hemicellulose at ambient temperature [103]. 

Alkaline reagents cause changes in the crystalline and morphological structure of cellulose. As a result, hydrolysis of the polysaccharide chain together with crystals saponification occurs [95,101,102]. Consequently, the biomass density decreases and the availability of monosugars increase at further hydrolysis stages [58,61]. 

Singh and Trivedi have shown that application of 3% NaOH results in higher efficiency of lignocellulose hydrolysis than using potassium or calcium hydroxide (temperature of approximately 70 °C) at the same concentrations [92]. Differences in the hydrolysis efficiency, i.e., the differences in the glucose concentration after hydrolysis between the above listed reagents may equal up to 50%. It is also stated that the level of degradation processes within the monosugar molecules constituting cellulose is lower for alkaline than for acid hydrolysis [92]. Furthermore, hydroxides may be recovered and recycled. According to Sun et al., the highest efficiency of wheat straw delignification occurs when 1.5% NaOH is used at a temperature of approximately 55–65 °C [16,65].

Haque et al. have shown that it is possible to produce the bioethanol precursor from barley straw using 2% NaOH at the boiling temperature. NaOH pretreatment resulted in external cell structure damage and increased the exposure of the internal cell structure. The external surface area increased. Consequently, it was stated, that a maximum of 84.8% removal of lignin and 79.5% removal of hemicelluloses may be achieved. A maximum reducing sugar yield of 86.5% was achieved during the following enzymatic hydrolysis process [152].

The efficiency of alkaline pretreatment depends on the lignin content in the biomass. It is known that alkaline pretreatment may be effectively applied to agricultural waste, e.g., wheat straw [8,34]. To decrease the high cost of chemical reagents, it is proposed to use ammonia or calcium hydroxide, which may be easily recovered, i.e., via chemical precipitation [51,98,105]. It was observed, that the monosugar concentration after corn straw pretreatment is higher for the shorter process time (1–3 h) at 85–135 °C (pressure below 10 Ba), than for the longer process time (up to 24 h), at lower temperature in the range of 50–65 °C. It was shown that the alkaline pretreatment of pretreated corn straw results in a monosugars concentration 10 times higher than compared to untreated straw [51,94,98]. The authors of the mentioned papers proposed a following correlation: the better the cellulose exposure to reagents is, the higher the enzymatic digestibility of the biomass is. The digestibility of the lignocellulosic biomass depends on the crystal size of polysaccharides but also on the efficiency of the non-enzyme-specific biomass components elimination. At the same time, reducing the sugars elimination is desired, as the sugars are substrates for the fermentation process and their concentration should be as high as possible.

Alkaline pretreatment enables extensive delignification and deacetylation [94,98]. Lignin elimination precedes the removal of inactive adsorption sites. The cellulose and hemicellulose availability by the enzymes increases. Chunk et al. investigated the hydrolysis of corn straw with calcium hydroxide (0.5 g Ca(OH)_2_ per 1 g of biomass) [98]. The reaction was carried out under air or nitrogen at temperatures of 25, 35, 45 and 55 °C. At 55 °C, the results of one-week digestibility were similar for oxidizing and non-oxidizing conditions (removal of approximately 90% of the acetyl groups). The highest efficiency of delignification was obtained at 55 °C with non-oxidizing conditions. The cellulose crystallization ratio did not change with the mentioned conditions. It is known that hemicellulose deacetylation reduces the steric hindrance for hydrolytic enzymes and increases the digestibility of carbohydrates [37,69].

Pretreatment of the following biomass types with calcium hydroxide was investigated: wheat straw (85 °C, 3 h), poplar wood (150 °C, 6 h, 14 Ba, oxidizing conditions), switchgrass (100 °C, 2 h) and corn straw (100 °C, 13 h) [106]. It was found that an optimal concentration of sodium hydroxide for hydrolysis of the mentioned lignocellulose materials (120 °C, 4 h) is 0.075 g Ca(OH)_2_ per 1 g of dry biomass. Additionally, the conversion of polysaccharides to monosugars is almost complete, in the case of corn straw. 

Application of sodium hydroxides to lignocellulose materials causes biomass swelling that leads to an increase in the specific area and a decrease in the polymerization and crystallinity ratios, separation of the structural bonds between lignin and carbohydrates, and an alteration of the lignocelluloses structure. The biomass digestibility of hard wood increases by approximately 14–55% after alkaline pretreatment with NaOH. The lignin content decreases simultaneously [99,106]. Alkaline pretreatment with NaOH is also efficient for straw with a low content of lignin (10–18%) [99]. Simultaneous pretreatment of lignocellulose materials with gamma radiation and hydrolysis with 2% NaOH was investigated [90]. The glucose conversion efficiency increased by 20% when corn straw was processed, by 3.5% for manioc bark and 2.5% for nut shells, compared to non-radiated samples.

Investigations of switchgrass hydrolysis with 1% NaOH followed by microwave radiation or conventional heating found an increase in the digestibility of biomass [107]. It was found that addition of ammonia during lignocellulose pretreatment increases the delignification efficiency [91]. The delignification efficiency was approximately 60–80% for corn cobs and 65–85% for switchgrass [3,91].

An alkaline pretreatment process based on the green liquor (GL), consisting of sodium carbonate and sodium sulfide, was developed for wood chips [153]. The GL pretreatment tends to selectively remove the lignin and leave both hemicellulose and cellulose fractions in the pulp. The moderate alkaline pretreatment helps to increase the selectivity of delignification, to disrupt the structure of the raw material and to expose the cellulose to enzymatic attack. Gu et al. obtained the highest efficiency of delignification (45% lignin removal) under optimized condition (8% total alkali, 40% sulfidity, 140 °C). Furthermore, approximately 70% of the original polysaccharides were converted into fermentable sugars in the subsequent enzymatic hydrolysis. The result indicates that green liquor can be used for the pretreatment on corn cobs to improve the enzymatic saccharification. 

Unlike acid pretreatment, GL pretreatment produces no toxic by-products, such as furfural or acetic acid, which are inhibitors in the fermentation stage and cause corrosion in the equipment. It is also interesting to reduce the production cost through integrating ethanol production with a combined heat and power plant or with a pulp and paper mill [153].

#### 3.2.3. Oxidizing Pretreatment of Lignocellulose Biomass

Oxidizing pretreatment consists of the application of peroxides (i.e., hydrogen peroxide) or alcohol solutions of peracids (i.e., peracetic acid). Oxidizing reagents dissolve lignin and amorphous cellulose, while hemicellulose undergoes dissolution only after disjoining from the biopolymer. Crystalline cellulose does not dissolve [103]. The following processes occur during the oxidizing pretreatment: electrophilic substitution, site chains dislocation and aryl-alkyl bonding cleavage [37]. 

Alkaline hydrogen peroxide (H_2_O_2_) is widely used in the paper industry as a whitening agent and for lignin and xylan removal. It is a very mild hydrolyzing agent because it does not leave any pollution in the biomass and it decomposes to water and oxygen. Moreover, no waste by-products of pentoses and hexoses decomposition are formed in its presence, which is a problem during acid hydrolysis [109]. Oxidizing pretreatment decreases the biomass recalcitrance and hydrogen peroxide reacts only with aliphatic biopolymer fragments. Alkaline conditions favor the separation of lignin from the lignocellulose structure, due to the presence of the cumene anion [50]. 

The hydrogen peroxide application requires special processing conditions. The reagent is instable in alkaline conditions and undergoes easy decomposition when transition metals are present (i.e., Mn, Fe, Cu). Oxidation reactions with H_2_O_2_ involve interactions of hydroxyl radicals, resulting from hydrogen peroxide degradation with anionic groups, leading to lignin oxidation and degradation. The highest pH value enabling efficient alkaline pretreatment with H_2_O_2_ is 11.5. The lowest applicable hydrogen peroxide concentration is 1% with a mass proportion between H_2_O_2_ and biomass equal to 1:4 [37].

Hydrolysis of wheat straw with 1% H_2_O_2_ (25 °C, 18–24 h) results in 50% delignification and partial dissolution of hemicellulose. When the pH of hydrolysis is approximately 11.5, almost complete delignification is possible. However, increasing the pH to 12.5 does not increase the digestibility of lignocellulose when alkaline H_2_O_2_ is used. The optimal dose of H_2_O_2_ is 2.15% (*v*/*v*) and the optimal temperature for biomass hydrolysis is 35 °C [109]. 

Peracetic acid is a strong hydrolyzing agent, enabling selective delignification with limited loses of carbohydrates. The hydrolysis efficiency and the digestibility of cellulose may increase up to 98% when peracetic acid is used for the biomass pretreatment in combination with NaOH [37].

Another biomass pretreatment method is ozonolysis. It is an efficient, but rather expensive method of hydrolysis. Ozone acts as a strong oxidizing agent that removes the lignin and partially removes the hemicellulose. It is a result of oxidation, cleavage of hydrogen bonds, as well as swelling and decomposition of biomass components. Cellulose is not affected by ozonolysis, as there is no branching in its structure. Ozonolysis increases the efficiency of enzymatic treatment of biomass by approximately fivefold [34]. No toxic hydrolysis by-products are formed when ozonolysis is used. It was found that ozonolysis may selectively degrade lignin [37,51,108]. In the case of corn straw, the lignin content decreased from 29% to 8% with ozonolysis. At the same time, hemicellulose structure is slightly altered, and cellulose structure is not changed.

Mild process conditions (ambient temperature, normal pressure) are the main advantages of ozonolysis. Additionally, ozone may be easily decomposed, i.e., via increasing the temperature of catalysis. However, the products of ozonolysis may contain fractions of oxalic acid, formic aid, glycerin acid, malonic acid, or succinic acid. It may be a result of the presence of the mentioned substances in wet biomass, e.g., in poplar [3,70,91]. Ozone does not oxidize the acids mentioned above.

#### 3.2.4. Lignocellulose Biomass Pretreatment with Ionic Liquids

Ionic liquids (ILS) are organic solvents existing as ion pairs. The melting temperature of ILS is below 100 °C. ILS are highly persistent, polar, and thermally stable single component substances [6,110,111]. The most popular ILS is imidazole salts.

Amimcl (1-allyl-3-methylimidazolium chloride) and Bmimcl (1-butyl-3-methylimidazolium chloride) may be effectively applied for cellulose dissolution when the temperature is below 100 °C [71,112,154]. It is probable that the mechanism of cellulose dissolution consists of the alteration of lignocellulose crosslinking by hydrogen bonds, which results in an increase in the biomass digestibility [70,73,110,112]. 

The cellulose fraction may be recovered by the addition of water, ethanol, or acetone. The regeneration process could significantly influence the crystallinity and digestibility of cellulose. Microcrystalline cellulose (MCC) was regenerated from ionic liquid 1-butyl-3-methylimidazolium chloride. For cellulose regenerated from acetone and wet regenerated cellulose from water, more than 90% of the cellulose could be converted into glucose within 4 h. The conversion ratio for the cellulose regenerated from water and dried in a freeze drier was approximately 90% after 40 h, whereas 48.5% of the conversion ratio was obtained for MCC after 96 h [154]. ILS may be reused after recovery via pervaporation, reverse osmosis, salting out or ion exchange. 

Biomass recalcitrance to hydrolysis may be overcome by the processes of COSLIF (Cellulose Solvent and Organic Solvent-based Lignocellulose Fractionation) or SPORL (Sulfite Pretreatment to Overcome Recalcitrance of Lignocellulose). Application of ILS enables the separation of cellulose from hemicellulose and lignin. Amimcl dissolves cellulose up to concentration of 10% (*w*/*w*) at 50–100 °C. The degree of cellulose may be increased to 25% (*w*/*w*) by simultaneously applying ILS and microwaves [74]. Emimcl dissolves cellulose up to 39% (*w*/*w*) at 105 °C [3].

ILS are regarded as environmentally friendly substances, mainly due to their low volatility. On the other hand, ILS are rather expensive solvents, especially when industrial amounts are considered. Very few results of biomass pretreatment with ILS have been found.

Experiments conducted under the different temperature conditions of 110, 120, and 140 °C for up to 5 h examined the influence of water content on hydrolysis yield. The maximum yield of reducing sugars were obtained when the process temperature of 110 °C was used for 3 h and the weight ratio of ionic liquid to the water was 20:1. Application of ionic liquid [Bmim]Cl in combination with an aqueous solution of the catalyst Fe_3_O_4_ C-SO_3_H also showed better accessibility of the interfacial area [155]. When ionic liquid [Hnmp]Cl and [Hnmp]CH_3_SO_3_ were applied at a temperature of 90 °C for 30 min, the recovery of lignin from maize stalks reached 85.94% [156].

#### 3.2.5. Pretreatment of Lignocellulosic Biomass with Organic Solvents and Other Chemicals

Cellulose pretreatment may be realized with mixtures containing organic solvents, i.e., LiCl/DMAc, NaOH/urea, carboxymethylcelluloses, of different polymerization degrees. According to Guo et al., a crucial step during the lignocellulosic biomass pretreatment is the cleavage of hydrogen bonds between the hydroxyl group OH-2 and oxygen O-6 in polysaccharides [75]. It may be achieved at elevated temperatures (100–250 °C). Application of acetone prior to applying the organic solvents increases the solubility of cellulose [76]. 

Popular organic solvents include different reagents, i.e., low boiling point substances (ethanol), glycerin, tetrahydrofurfuric alcohol, ethers, ketones, phenols, organic acids and dimethylsulfoxide [34,51,77]. The mentioned substances may be applied as water soluble mixtures, optionally containing catalysts, i.e., inorganic acids (HCl, H_2_SO_4_), or organic acids (oxalic acid, acetylsalicylic or salicylic acid), that aid in the dissolution of hemicellulose and lignin. At specified conditions, almost complete delignification, as well as highly efficient hemicellulose hydrolysis are possible, as a result of the following two independent mechanisms:

- Hydrolysis of internal lignin bonds and 4-*O*-methylglucuronic bonds between lignin and hemicellulose;
- Hydrolysis of glyosidic bonds in hemicellulose and partially in cellulose [76,80,100].

At temperatures higher than 185 °C, the impact of catalyst presence decreases due to the formation of organic acids as a result of biomass decomposition. These organic acids act as an autocatalysis [79]. 

Delignification kinetics depends on the type of solvent and biomass material [3,37]. Organic solvents must be dried and evaporated after hydrolysis completion because of economic, as well as process reasons (elimination of the negative impact on microbe development, enzymatic hydrolysis, and fermentation) [78,79,80,100]. The products of hydrolysis with the addition of organic solvents are of high quality and purity and have low sulfur content. Such hydrolysis products find application in heat production and as alternatives to other polymers, i.e., to phenol resins or polyurethane [71,79]. 

Application of organic solvents is usually accompanied by other pretreatment methods [37,48,79,80]. The disadvantage of organic solvents application is the possibility of side-reactions taking place in the solution, resulting in an increase of inhibitors, i.e., furfural concentration that inhibits the fermentation process. Moreover, the presence of volatile organic compounds may pose a fire risk and is rather inconvenient, in terms of the environmental as well as safety aspects.

Kabir et al. applied methanol, ethanol, and acetic acid, separately, and with additions of acidic catalysts. Pretreatment was carried out at a temperature of 190 °C, 50% (*v*/*v*) of organic solvent for 60 min. The use of organic solvents resulted in a decrease in the content of lignin and hemicellulose in the plant material compared to the untreated material. The addition of sulfuric acid as a catalyst allowed a further reduction of the hemicellulose content but increased the content of acid-insoluble lignin compared to samples treated with organic solvents without the addition of acid. Depending on the solvents and the catalyst used, the cellulose content in the material is in the range of 31.2–43%, which, compared to the untreated material (in which the cellulose is 22.3%), is a considerable increase [157].

### 3.3. Physicochemical Pretreatment of Lignocellulosic Biomass

#### 3.3.1. Biomass Pretreatment with Hot Water

Biomass pretreatment with hot water, i.e., wet air oxidation together with/or steam explosion exclusively is the most investigated physicochemical method of biomass pretreatment. These methods use the differences in the thermal stabilities of the major components of lignocellulosic materials. Because severe degradation of the cellulose structure occurs at temperatures higher than 240 °C, hemicelluloses and partial lignin can be removed easier than cellulose [154,158]. As the pretreatment temperature reaches 240 °C, the cellulose was partially degraded, and the crystallinity of the cellulose is reduced [158]. Application of steam explosion at 160–240 °C (0.7–4.8 MPa) results in an increase in the hydrolysis efficiency by approximately 220%, in the case of biogas production [78,114]. 

Pretreatment with hot water may be realized as follows. The fragmented lignocellulosic material is exposed to saturated steam at 160–240 °C under the pressure of 0.7–4.8 MPa. The pressure in the reactor is retained from several seconds to several minutes. Under these conditions, hemicellulose hydrolysis occurs and above 180 °C, lignin hydrolysis also occurs. Then, the reaction vessel is quickly decompressed and left to cool down. As a result, dissolved hemicellulose and lignin are transferred to the liquid phase, while cellulose remains as a solid. The biomass digestibility increases, as cellulose is more susceptible to hydrolytic enzymes. At the same time, partial hydrolysis of cellulose occurs as a result of acetic acid formation [3,37]. Hemicellulose hydrolysis releases glucose and xylose, which is called autohydrolysis. A high degree of hydrolysis (up to 95%) may be obtained at 270 °C (process time 1 min.). 

Xiao et al. pretreated bamboo residues with hot water at 140–200 °C for different times (10–120 min) [159]. The degradation efficiency of lignin showed a slight increase with the increase in temperature and time. However, a significant amount of lignin remained in the residues after the hydrothermal pretreatment. The content of lignin (acid-soluble lignin and acid-insoluble lignin) in the residues increased from 25.9% to 41.1% with the increase of temperature and reaction time. 

The cellulose and hemicelluloses in the biomass might be partially converted into pseudo-lignin under the hydrothermal conditions, which resulted in the increase of acid-insoluble lignin. The pseudo-lignin, which mainly consisted of carbonyl, carboxylic, aromatic, and aliphatic structures, significantly inhibits enzymatic hydrolysis of cellulose because of its redeposition on the surface of the biomass residues [160]. The relocation of lignin during the hydrothermal pretreatment may negatively affect enzymatic hydrolysis and reduce the yield of glucose [161,162,163]. 

However, side-products of sugars decomposition are formed during hot water pretreatment. These side-products, i.e., furfural from pentoses decomposition, acetic acid from hydrolysis of the acetyl groups from the hemicellulosic polymers and 5-hydroxymethyl furfural HMF from hexoses decomposition, inhibit the fermentation process [158]. The mentioned inconvenience may be omitted by applying a lower temperature and longer process time (i.e., 190 °C, 10 min.). Furthermore, application of an acid as a catalyst increases the monomeric sugars formation from hemicellulose by approximately 24%. Additionally, the catalyst application decreases the formation of fermentation inhibitors and increases the enzymatic hydrolysis efficiency.

Hot water pretreatment reduces chemical reagents usage. It is also a rather economic and highly efficient process. Because lignin is only partially hydrolyzed, there is a possibility of condensation and degradation of xylan. Therefore, wet air oxidation can be applied in the pretreatment of hard wood and agricultural waste; however, it is not efficient for conifers. Efficient pretreatment of hard wood may be realized via purging of hot medium through the immobilized lignocellulosic bed at pH 4–7 [4].

Sun et al. investigated a two-step pretreatment using hydrothermal pretreatment at various temperatures and alkali fractionation on eucalyptus fiber. Compared to the hydrothermal pretreatment, the hydrothermal conditions associated with alkaline treatments significantly removed hemicelluloses and lignin. As a result, the enzymatic hydrolysis rate in the cellulose-rich fractions increased from 3.7 to 9.2 times. The enzymatic hydrolysis rate increased from 1.1 to 8.5 times as the hydrothermal pretreatment temperature increased from 100–240 °C. An optimum pretreatment condition was found to be hydrothermal pretreatment at 180 °C for 30 min and alkali fractionation with 2% NaOH at 90 °C for 2.5 h, in which 66.3% of the cellulose was converted into glucose by enzymatic hydrolysis. In these conditions, the consumption of energy was the lowest and the lignin removal was the highest achieved [158]. 

Amiri et al. applied the autohydrolysis process using steam and high-pressure conditions with 180 °C as a pretreatment step before enzymatic hydrolysis. The main products of the hydrolysis were composed of monosugars and oligosugars from the degradation of hemicellulose. Autohydrolysis causes swelling of the dense structure of wood, with the recesses larger than 20 microns. As a result, the accessibility of hydrolytic enzymes increases [155,164].

#### 3.3.2. Pretreatment with Carbon Dioxide

The mechanism of biomass pretreatment using carbon dioxide explosion is similar to steam explosion [34]. Carbon dioxide explosion uses supercritical CO_2_ (SC-CO_2_) to aid the biomass digestibility. SC-CO_2_ or high-pressure CO_2_ (up to 28 MPa) is supplied to the biomass placed in a high-pressure vessel [47,95,98]. The reaction vessel is heated to a specified temperature (i.e., 200 °C, process time of several minutes) [37,47]. Under the mentioned conditions, CO_2_ penetrates the biomass. Additionally, it is believed that carbonic acid is formed as a result of CO_2_ dissolution in water. Carbonic acid increases the rate of hydrolysis, especially hemicellulose hydrolysis [34,47]. The decompression of the reaction system causes alteration and fragmentation of the biomass structure, which increases its specific area [95]. 

Pretreatment with carbon dioxide increases the efficiency of cellulose hydrolysis to glucose by approximately 70% compared to the untreated biomass [37]. Moreover, biomass treated with CO_2_ is more vulnerable to hydrolytic enzymes [34]. 

Pretreatment with carbon dioxide is efficient with respect to wet biomass only. The higher the moisture content, the better the hydrolysis efficiency [47,98]. The advantages of the discussed method include the low cost of CO_2_, no toxic substances formed, the possibility of process realization at mild temperatures (35 °C at 7.3 MPa) as well as the treatment possibility of biomass having high solids content [37,47]. The main disadvantage is related to the high cost of the high-pressure equipment. 

#### 3.3.3. Biomass Pretreatment with Ammonia

Biomass pretreatment with ammonia is realized at elevated temperature and pressure. The most popular methods are AFEX and ARP (Ammonia Recovery Process). 

The AFEX method consists of forming a biomass suspension in anhydrous ammonia (1–2 kg of ammonia per 1 kg of dry biomass). The process is carried out at 60–90 °C with pressure above 3 MPa for 10–60 min. The mixture of biomass and ammonia is heated to the desired temperature for approximately 30 min. The suspension is kept at the specified temperature and pressure for approximately 5 min and then the system undergoes a sudden decompression. As a result, the ammonia undergoes fast evaporation and the system cools down. The pressure variations cause swelling of the cellulose and increases its specific area. Such a phenomenon is described as a change in the crystal cellulose structure [105]. During the AFEX process, a small portion of hemicellulose undergoes dissolution to oligomeric products and the lignin structure changes. The AFEX method may considerably increase the saccharification of plants, as well as agricultural and urban wastes [105]. 

During the ARP, the ammonia solution percolates through the packed bed. The ammonia aqueous solution (5–15%) flows through the biomass bed (10 mesh granulation), in the column reactor, at an elevated temperature (150–180 °C). The volumetric flow rate of ammonia is approximately 1–5 mL/min and the retention time of the solution in the reactor is approximately 10–90 min. Then, the solution is recycled or recovered [98,106]. At such conditions, the hydrolysis of lignin and hemicellulose occurs and the degree of cellulose crystallinity decreases. Pretreatment methods using ammonia may also be efficient with respect to hard wood [105]. The exhausted ammonia solution may be recovered and recycled, while the obtained hydrolysates do not contain fermentation-inhibiting substances [79]. Moreover, minimalization of fermentation inhibitors formation results from mild process conditions (temperature of approximately 100 °C, pH below 12, relatively short retention time). 

Ammonia is highly efficient with respect to lignin, enabling even up to 70–85% delignification of corn straw in 20 min and approximately 40–60% removal of hemicelluloses. At the same time, the cellulose structure is conserved [98]. However, pretreatment with ammonia is not efficient for biomass with high lignin content, i.e., biomass form paper production or conifers [48,80]. Additionally, the poisonous ammonia fumes generated during process realization causes safety and environmental concerns. 

### 3.4. Enzymatic Methods of Lignocellulosic Biomass Treatment

On completion of the pretreatment step using mechanical, physical, chemical, or physico-chemical methods as well as possibly efficient delignification, deacetylation and partial release of simple sugars, hydrolysates are treated with biochemical methods. Enzymatic hydrolysis aims at the release of simple sugars from crystalline cellulose and hemicellulose. The obtained monosugars are then fermented. Thus, the efficiency of enzymatic hydrolysis plays a key role in the efficient conversion of biomass to the desired products [3,37]. 

It is crucial to select a pretreatment method that enables high saccharification efficiency while the inhibitors formation, energy consumption and materials usage are reduced to a minimum. Due to the pretreatment step, the efficiency of enzymatic hydrolysis of lignocellulosic material is higher and the process is more cost-effective. The resulting glucose is a carbon source for many fermenting organisms.

Cellulose and hemicellulose conversion is catalyzed by cellulases and hemicelluloses. Cellulose hydrolysis may be realized with the application of the following enzymes: endoglucanases (hydrolysis of internal beta-1,4-glicosidic bonds), exoglycanases (removal of monomers and dimers from the end of the glucose chain) and glycosylases (hydrolyzes glucose dimers). Cellulose degradation is a complex process, as cellulose microfibers are stabilized by internal and external hydrogen bonds and are surrounded by hemicellulose polysaccharides, i.e., mannans and xylans [118,119]. Cellulases catalyze the decomposition of cellulose to fermenting sugars [3]. 

Cellulose hydrolysis begins with the adsorption of cellulases on the cellulose surface and then the degradation of cellulose to simple sugars. Desorption of cellulases from the biomass surface also occurs. Hemicellulose may be decomposed by different enzymes. Xylan is hydrolyzed with endoxylanases and beta-xylosydases, which causes xylan degradation to xyloolygosaccharides. Enzymes, such as α-glucuronidase, α-arabinofuranosidase and acetoxylan esterase, are responsible for cleavage of side groups and chains of heteroxylan. β-mannanase and β-mannosidase are responsible for glucomannan hydrolysis [3]. 

The main commercial source of cellulases are filamentous fungi and their cellulolytic strains called *Tichoderma viride*, *Tichoderma longibrachiatum*, *Tichoderma reesei* and *Penicillum* [118]. *Aspergillus* sp. is applied, with a source of β-glucosidase, xylanase and xyloglucanase. There are also species of aerobic *Actinomycetes* that form systems of enzymatic nanocomplexes, such as *Cellulomonas*, *Thermobifida* and anaerobics, that generate complexes, i.e., cellulosomes of *Clostridium* and *Ruminococus* [35,112,119]. *Trichoderma reesei* is the best-known source of cellulose, cellobiohydrolase, endoglucanase and β-glucosidase [68]. The digestibility of cellulose and hemicelluloses, beside the quality of hydrolyzing enzyme, depends upon the pH, temperature, process time, porosity, crystallinity degree, as well as the cellulose content [119,120].

Enzymatic synergism, i.e., an increase of hydrolysis efficiency due to simultaneous action of two or more enzymes, depends on the substrate type, crystallinity degree and enzymes’ specific features, governing the interactions on reducing and non-reducing polysaccharide sites [71,79,80,121,122,123,124]. The impact of pretreatment on the interactions between cellulases and lignocellulosic complex is not well described in the literature. According to Nomanbhay et al., the most efficient method of hard wood pretreatment (red oak, fir) includes a combined application of acid hydrolysis (0.1% H_2_SO_4_), enzymatic hydrolysis and vibrational milling [117]. Pretreatment of cotton in acetone solution enables the conversion of cellulose up to 98.6% [94]. Samayan and Schall investigated hydrolysis of lignocellulose pretreated with ILS [118]. They reached a complete conversion of cellulose to glucose after 24 h, when a mixture of enzymes was used.

Proportions of cellulases applied in the investigations of the digestibility of lignocellulosic materials differ widely. Bothwell et al. showed that the highest digestibility of cellulose is possible when a mixture of cellobiohydrolase and endoglucanase in a proportion of 1:3 is used [118]. The proportion of the same substances was set as 98.75–1.25% by Boisset et at. [122], 8.1:1 by Ramirez et al. [125], 17:1 by Berger et al. [126] and 10:1 by Jung et al. [106]. It is known that optimal proportions of enzymes depend on the pretreatment method and the presence of enzyme-inhibiting substances.

Three mechanisms of synergism are identified in terms of hemicellulase [127]. These are homeosynergism, heterosynergism and antagonism. Homeosynergism occurs between enzymes selective to the main chain saccharides. Heterosynergism occurs between enzymes selective to side chains, while antagonism occurs when one enzyme blocks the action of the other enzyme, i.e., via elimination an enzyme-specific substituent from the chain. 

*Clostridium stercorarium* produces eight different enzymes; however, only three of them are required for degradation of the sugar complex [128]. According to Vardakou et al., the activity of enzymes decomposing the main chain is higher when the hydrolysis is divided into two steps [129]. It is convenient when enzymes cutting links in side chains and branches are used in the first step. However, Sorensen et al. stated that such an approach limits the digestibility because of the removal of enzyme-specific substituents [130]. 

Xylanases may have a large active center that is responsible for cleavage of main chains in non-substituted regions or smaller active centers that cleave main chains close to substituents [129]. Several synergistic interactions have been identified in the case of hemicellulases. Both the acetyloxylan esterase and endoxylanase present increased the production of reducing sugars and the release of acetate residues from xylan chain [131]. According to Kam et al., acetyloxylan esterase is responsible for xylan deacetylation and acts independently on xylanase [132]. 

In the presence of xylanases with small active centers, the production of ferulic acid is increased in the absence of acetyloxylan esterase [129]. An increase of bioconversion efficiency was also noted for the mixture of *p*-xylosidase and acetyloxylan esterase [133], or the mixture of α-l-arabinofuranosidase and exoxylanase [134], or the mixture of endomannose and endoxylanase [135] when sugar cane extrusion materials are processed. It is known that xylan blocks the access of cellulases to cellulose and an addition of xylanase to the enzymatic mixture increases the efficiency of cellulose hydrolysis. However, it is not yet known whether the same rules may be applied in terms of mannan and pectines and whether these substrates block the cellulase access to cellulose [107,132,135,136].

Biomass pretreatment research compared the performance of basic hydrolysis using *Tetraselmis suecica* and microalgae *Chlorella* sp. and the following basic catalysts KOH, NaOH, NH_4_OH and Na_2_CO_3_. The process was carried out at 90 °C for 75 min, while the concentration of the catalyst used was up to 0.3 M. The most effective catalyst was potassium hydroxide, and the maximum yield of sugars reduction reached even 81 mg/1 g of dry biomass [165].

Song et al. examined the synergistic effect of cellulase and xylanase-based enzymatic hydrolysis of three different plant materials (corn cob, corn stover and rice straw) using cellulase and xylanase together in comparison with the use of these enzymes separately. The conversion efficiency of the reducing sugars, derived from corn stover using a mixture of enzymes was 48.5%, and for the rice straw, it was 31.1% [166].

### 3.5. Lignocellulosic Biomass Treatment with Microorganisms

Microbial methods of biomass pretreatment are based on wood-decomposing fungi. Such fungi produce enzymes able to depolymerize or cleave lignin, cellulose, and hemicellulose. Wood-rot fungi acts via the chemical decomposition of plant cell walls, leading to color change and wood degradation. Fungi may attack all forms of wood, i.e., growing trees, timber wood, as well as wood furniture. Changes in the chemical composition, as well as the macroscopic view of wood are divided into the following three main groups of wood rot: brown rot (decomposition of cellulose and pentosanes), white rot (decomposition of lignin), and corrosive rot (decomposition of all components of wood) [98]. 

Rot can produce several enzymes, i.e., lignin peroxidase, polyphenol oxidase and magnesium-dependent peroxidase. Investigations regarding the application of rot generated by *Phanerochaete chrysosporium*, *Phlebia radiate*, *Dichmitus squalens*, *Rigidosporus lignosus* and *Jungua separabilima* show that the rot can selectively cause the hydrolysis of lignocellulose and depolymerization; however, the process is time-consuming and lasts at least one week [137,138]. 

Enzymes produced by white rot may be divided into three main fractions. The first fraction includes cellulolytic enzymes (endo-1,4-β-glucanase, exo-1,4-β-gluconase and glucohydrolases), hemicellulases (i.e., endo-1,4-β-xylanases, β-xylosidases, galactoglucomannazes or galactosides). The second fraction includes enzymes responsible for lignin degradation, of which lignin peroxidase, dioxygenases, peroxydismutases and glyoxal oxidases are the most important. The third group includes enzymes specifically interacting with lignosaccharides (glucose oxidase, piranose oxidase, oxyreductase and cellobiase). The mentioned enzymes may act separately or synergistically. However, their isolation and purification are expensive and complicated. Thus, they are mainly used as mixtures [137].

White rot is applied for degradation of rye straw. The most selective fungi species in this group are *Phanerochaete chrysosporium*, *Cyathus stercoreus* and *Pleurotus* spp. [139]. In practice, enzymatic extracts of the mentioned species are used. It was shown that the structural changes of lignin and hemicellulose are dependent on the species [138]. Moreover, losses of hemicelluloses were observed. The lowest hemicellulose losses were noted for *Pleurotus erynii* and *Phanerochaeta chrysosporium*, where at least two lignolytic enzymes were present.

Lignolytic activity of white rot is correlated with peroxidase activity in the liquid culture of *Phanerochaeta chrysosporium*. All isozymes isolated together with peroxidase are more persistent [139]. The fermentation processes realized with enzymatic extracts are more efficient than those performed with microbiological cultures. This is because the removal of cell walls is crucial for the fermentation process and only lignin-modifying species are of industrial importance.

Lignin is a fraction responsible for biomass recalcitrance in the case of fermentation processes. However, even after the removal of lignin, it is required to increase the digestibility of other sugar fractions, i.e., cellulose and hemicellulose. Brown rot fungi are applied to fulfil this aim. Brown rot can depolymerize amorphous cellulose [140,141]. The mechanism of brown rot action consists of the modification of the lignin structure by means of demethylation as well as oxidation and degradation of hemicellulose and cellulose. The side product of such hydrolysis is a brown lignin suspension in oligosaccharide solution. The main strain of brown rot is *Gloeophyllum trabeum*, which produces β-glucosidase, thermophilic xylanase, as well as cellulases and hemicellulases [141].

Soft rot fungi are *Ascomycota*, *Deuteromycota*, *Trichoderma reesei*, *Chaetomim* sp. and *Ceratocystis* sp. [35]. The above listed fungi species are especially effective towards wood with high moisture content. Soft rot acts via secondary erosion of cell walls followed by hemicellulose-cellulose complex decomposition. As a result, the lignin fraction is eliminated, and polysaccharides are decomposed to monosaccharides. However, monosaccharide consumption by soft rot is rather high.

The main advantages of fungi application to lignin depolymerization are high selectivity and efficiency. Unfortunately, the microbial pretreatment is often cost-intensive in terms of money and time and therefore is not often used at a large scale [47]. 

## 4. Perspectives in the Biomass Pretreatment Sector

Production of biofuel from renewable resources, such as biomass and biofiber, gained great attention to partially replace fossil fuels in the future, as rapid population growth leads to environmental problems. Issues concerning sustainability are more often concerned when analyzing new potential energy sources [167].

Together with the growing demand for energy and energy prices and the implementation of policies in the field of global warming reduction, development of energy production technology from renewable raw materials is defined as strategic priority. Renewable energy is not only a source of energy, but also a tool to solve problems related to energy security, climate change, environmental friendliness, and sustainable development. Petroleum has the largest share of carbon dioxide emissions, air pollution and acid rain, which in turn have the largest impact on global climate change. Therefore, renewable energy sources as an alternative to conventional fossil fuels have been presented as the main energy supplier in the future. Biomass combined with the capture and storage of carbon dioxide is considered the only reliable method to reduce greenhouse gas emissions [168]. It is estimated that, for example, ethanol production from biomass has the potential to reduce greenhouse gas emissions by 86% [169]. 

The most important criterion for choosing a raw material for biofuel production is its availability, carbohydrate content, fermentability and cost. The biomass is the most abundant biopolymer on Earth and is a sustainable and cheap resource that can be turned into fuels and chemicals on a large scale. In addition, agricultural oil and sugar crops and their by-products, lignocellulosic products such as wood and wood waste, aquatic plants such as algae and water weeds, industrial or municipal waste, and animal waste are accepted as biomass sources. Lignocellulosic biomass is also attractive because it enables the development of products with new functionalities and molecules that would be less available or unavailable in fossil fuel-based technologies [168]. Many European countries as well as the United States, China and Brazil invest in the development of biotechnology, with particular emphasis on the conversion of biomass into bio-based industrial products, from the early 2000s. As a result of biological or chemical conversion of lignocellulosic biomass may be produced several products of interest for food, pharmaceutical and biofuels industries. The most important value-added products are organic acids (lactic, citric, acetic, succinic), glycerol, sorbitol, xylitol, 2,3-butylene glycol, fiber additives and hydrogels [40].

To effectively use lignocellulosic biomass is necessary to fractionate the biomass into its components by pretreatment. Despite the investigation and development of many methods, pretreatment is still one of the most expensive steps in the entire process of conversion of lignocellulosic biomass into biological products. The technoeconomic analysis of lignocellulosic ethanol production processes shows that the steps of pretreatment representing from 11–27% of total costs, depending of pretreatment method [170,171]. For the pretreatment process to be effective and economically viable, it must ensure: (i) high sugar yields for the fermentation step; (ii) low concentration of fermentation steps inhibitors; (iii) recovery of hemicellulose and lignin for later use in the production of valuable by-products; (iv) low demand of post-pretreatment operations such as washing and neutralization; and (v) minimal energy, chemicals and water inputs [54,168,172].

Each of the biomass pretreatment methods have specific disadvantages such as high cost, high energy consumption, less sugar degradation, environmental pollution, long reaction time or high requirement for reaction equipment corrosion resistance. The choice of the pretreatment technology depends on a variety of factors, such as the type of biomass or the value of by-products. It is not possible to choose the one pretreatment method that is preferred to use in commercial plants. However, methods dedicated for the specified type of lignocellulosic biomass could be indicated. The chemical pretreatment with sulfite-based chemicals is the most commonly used for degradation of woody biomass. Steam explosion has been recognized as one of the most efficient and cost-effective pretreatment technologies for agricultural residues, especially in processes scaled for commercial production.

Pretreatment step is still a challenge for obtaining cost-effective and competitive technology for large-scale conversion of lignocellulosic biomass into fermentable sugars with the low inhibitory concentration. An industrial technology for bioethanol production [173] from agricultural residues with the steam explosion method as the core technology was proposed. The effect of steam explosion of the corn stover was studied within a 50 m^3^ industrial reactor. After pretreatment 80% of hemicellulose was hydrolyzed and recovered and more than 90% of cellulose was left in the biomass [173].

Pretreatment using diluted acid is favorable for scale-up production of biofuels, because of the high efficiency to convert most of hemicellulose into soluble sugars and the use of cheap chemicals. However, the process generates inhibitors such as furfural and phenolic components. The efficiency of acid hydrolysis can be enhanced by optimizing pretreatment operation, conditions, and parameters such as temperature, pressure, retention time, solid/liquid ratio, and acid dosage

Silva et al. [174] studied the industrial-scale bioethanol production processes with three different pretreatment technologies: diluted acid pretreatment, liquid hot water pretreatment and AFEX pretreatment. Technoeconomic analysis showed that the process including liquid hot water pretreatment step turned as the most economically process.

The new approach to reduce the risk of technological problems occurring in pretreatment processes performed as a single operation, is to apply combined pretreatment including mechanical crushing-chemical, physical, or biological treatment, mechanical crushing-electronic radiation-alkali treatment, mechanical crushing–microwave–chemical processing, and mechanical crushing-chemical treatment-steam explosion [66]. The appropriate combination of several treatment methods, depending on the type of biomass, integrates the advantages of several individual pretreatment methods. In addition, this in turn can significantly improve the efficiency of enzymatic hydrolysis. Binod et al. [175] used combined microwave-acid, microwave-alkali, and microwave-alkali-acid pretreatment instead of conventional acid or alkaline pretreatment. It was found that combined microwave-alkali-acid treatment for short duration enhanced the fermentable sugar yield. Lai et al. [176] recommended low-energy ultrasonic-alkaline pretreatment to enhance the biodegradability of feed biomass. Liquid ionic/ultrasound pretreatment of bagasse, using biocompatible cholinium ionic liquid, showed the cellulose and hemicellulose saccharification percentages of 80% and 72%, respectively and a small inhibitory effect on cellulase [177].

In many cases, the integration of bioprocesses into the processing industry can be crucial to achieve rational energy efficiency and savings. For example, the spent cooking liquor in a craft pulp mill could partly be converted into biofuels or specialty cellulose products [169].

The total cost of producing biofuels from lignocellulosic biomass through fermentation can be lowered by wastewater management [178]. Wastewater from biorefinery stillage contains dissolved unused fermentable sugars and, mainly, lignin (74.1–79.0 wt. % of the biomass) [179]. The best use of this lignin is its use to produce high-value products or through direct combustion to supply industrial process energy [178]. However, fast pyrolysis has recently attracted researcher’s interest as it has the potential to convert lignin into to the high commercial value products such as bio-oil and bio-char [179].

The industrial application of a specific approach for the production of gaseous biofuels, requires a comprehensive analysis of its costs. Operating costs, variable and fixed expenses, and replacement costs should be taking into account the optimal method for biogas production. However, the commercial issues of the proposed procedures depend on the prices of fossil fuels and policy on biofuels.

In the field of biogas production, there are many installations producing biogas by anaerobic digestion, usually integrated with heat or energy generation. In case of biohydrogen, high cost and low hydrogen yields as well as relatively low operating fermentation broth concentration are still major challenges in the development of its production. Integrated technologies for biohydrogen production are proposed, taking into account the use of added-value products and co-generation of energy.

## 5. Conclusions

The application of waste lignocellulosic materials is a convenient method for the production of clean energy, as well as reducing of organic wastes. Biomass is converted to energy via fermentation processes. A crucial stage of fermentation is enzymatic hydrolysis of biomass during which the degradation of organic polymers to simple compounds occurs. Thus, enzymatic hydrolysis governs the course and the efficiency of fermentation and is the most expensive element of the fermentation process. The vulnerability of biomass to biodegradation depends on the degree of the material’s polymerization, moisture content, hemicelluloses and cellulose or porosity [180]. Also, the properties of the material being decomposed have a large influence on the rate of biodegradation: structure of biomass surface, thickness and shape, presence of crosslinking bonds, molecular weight, length of chains, hydrophobicity, hydrophilicity, cellulose crystallinity, biomass availability, lignin content. The most efficient method of hydrolysis combines the production of simple, easily fermenting substances together with the production of substances with complex molecular structures that may be used, i.e., for drugs production.

A side effect of lignocellulosic biomass pretreatment is the formation of by-products inhibiting the microbial and enzymatic biocatalysts. When technologies for biorefining of lignocellulose are transferred from laboratories to industry, the management of inhibition problems in feedstocks must be handled. Pretreatment of recalcitrant feedstocks, obtaining high product yields and high productivity could minimize the charges of enzymes and microorganisms to obtain high product titers [54]. Overcoming the physical and chemical barriers existing in the lignin carbohydrate structure requires a pretreatment method. Pretreatment is necessary to render most of the plant cell wall components for biomass conversion into valuable products, such as bioethanol or biohydrogen.

Pretreatment of lignocellulosic biomass is significant prior to enzymatic saccharification. Pretreatment of lignocellulosic biomass is significant prior to enzymatic saccharification. The conventional chemical pretreatment, using acids and bases, is regarded as a low-cost process. However current research directions indicate that by-products formed during chemical pretreatment must be purified and reused as value-added products. This type of approach may result in economic justification for the treatment of lignocellulosic biomass as a feed in bioconversion processes [149]. Biomass pretreatment is a crucial stage of lignocellulosic materials processing when biofuels and other bioproducts production is considered. The currently popular pretreatment methods are usually high energy consuming. Therefore, the development of efficient and cost-effective pretreatment methods and studies are of special importance from an industrial viewpoint.

Despite an increasing interest in lignocellulosic materials pretreatment, the published results of studies do not provide information regarding the influence of the structure and properties of the raw materials on the pretreatment efficiency. Additionally, the effects of process conditions, material’s composition or presence and concentration of by-products on the efficiency and the rate of enzymatic hydrolysis are rarely discussed. Therefore, investigation of possible mechanisms of changes occurring during the pretreatment together with experimental verification would be of high both scientific and practical value.

The authors of the present work believe that investigations currently performed will supply information regarding the abovementioned hydrolysis processes. The preliminary investigations imply that mechanical pretreatment, however indispensable in many cases, should be minimized. For example, steam explosion enables a direct pretreatment of biomass and could be applied without using mechanical size reduction. Additionally, steam explosion is one of the most cost-effective and the most efficient pretreatment technologies for agricultural residues. The chemical pretreatment should be applied in the case of wood processing.

The new approach to overcome disadvantages of pretreatment method performed as a single operation is the employ the combination of different methods. This approach leads to the integration of advantages of individual methods. Additionally, chemical pretreatment should be applied in the case of wood processing.

The selection and elaboration of technical analysis procedures for hydrolysis and fermentation processes, both for the liquid and gaseous phases is still a challenge in the field of bioenergy. Qualitative and quantitative information regarding the changes in the material’s and product’s composition is crucial for describing the process mechanism and kinetics.

Many years of research on the pretreatment of lignocellulosic biomass did not indicate a specific trend, which can be considered optimal in relation to the methods used and their subsequent impact on the bioconversion processes of the obtained hydrolysates. Preferred directions of future research may vary depending on the approach, mainly due to the very high potential of the obtained hydrolysates. It should be remembered that using hydrolysates exclusively as a source of sugar may be unprofitable from an economic point of view. increasingly often, attention is paid to by-products of hydrolysis and pretreatment, which are treated as added-value products. Therefore, using a well-chosen pretreatment method, may be crucial from the economic point of view. To use the raw material efficiently, it is necessary to analyze the potential economic and energy effects of the pre-treatment process. Consequently, for each biofuel production, optimization should be carried out, preferably in a two-step manner. The value-added products must be obtained with high purity, as they are most commonly formed as a result of the transformation of lignin and hemicellulose derivatives. Finally, the specific products, namely saccharides of cellulose and hemicellulose must be easily separated from other hydrolysis products to be used in bioconversion processes.

There are already known industrial chemical pretreatment methods, which provide high-digestible feedstock, recycling of wastewater and complete recovery and use of liquid and solid production waste; however, they can be used and optimized mainly for bioethanol [181]. The most promising direction of studies regarding biohydrogen and other biofuels production from lignocellulosic biomass is to conduct the production processes to obtain simple chemical compounds. Additionally, other useful organic products, i.e., drugs, beside biomass processing may be simultaneously generated. Such an approach is inevitable for both economic- and process-efficient treatment of lignocellulosic biomass [182].

Non-renewable fuels use is responsible for major environmental problems over time, such as global warming, climate change and health related problems. Along with global awareness about clean technology needs, research efforts have emerged intending to propose viable economically and environmentally clean alternatives.

## Figures and Tables

**Table 1:** Energetic potential of selected fuels.

Fuel	Energy Type	Energetic Value [MJ/kg]	Energetic Value [kWh/kg]	Applications	References
Hydrogen (compressed 500–700 Ba)	Chemical	120–142	39	rocket engines, automotive engines, grid storage and conversion	[26,27,28,29]
Gasoline	Chemical	47	13	automotive engines, power plants	[26,29,30]
Propane-butane gas	Chemical	45–46	13	cooking, home heating, automotive engines, lighter fluid	[26,29,30]
Heating oil	Chemical	40–42	11	home heating	[26,29,30]
Coal	Chemical	20–27	6–9	electric power plants, home heating	[26,29,30,31]
Firewood	Biological	19	6	electric power plants, home heating	[26,29,30,31]
Pellet	Biological	16.5–17.5	5–6	home heating	[26,29,30,31]
Biogas	Chemical	16.7–23	5–7	home heating	[26,29,30,31]
Lithium-ion Battery	Electrochemical	1.8	0.5	portable electronic devices, flashlights	[31]
Ethanol Fuel (E100)	Chemical	26	9	flex-fuel, racing, stoves, lighting	[29,30,31]
Tritium	Nuclear decay	583,500	162,000	electric power plants (nuclear reactors), industrial process heat	[30]

**Table 2:** Average shares of main components of selected lignocellulosic biomass materials in selected raw materials [39,40,41].

Biomass Type	Cellulose	Hemicellulose	Lignin
	% *w*/*w*		
Barley straw	33.8	21.9	13.8
Corn cobs	35.0	16.8	7.0
Cotton residues	58.5	14.4	21.5
Rice residues	36.2	19.0	9.9
Sugar cane	40.0	27.0	10.0
Wheat straw	32.9	24.0	8.9

**Table 3:** Methods of lignocellulose pretreatment.

Method	Type of Pretreatment	Mechanism of Action	References
Physical	Fragmentation (hacking, grinding, milling, rolling)	Fragmentation of lignocellulosic chain into smaller parts, exposing of lignocelluloses structure to reagents in further treatment steps	[9,34,37,48,49,68,75,81,82,83]
	Microwave radiation	Reduction of cellulose crystal structure	[3,4,6,10,23,84,85,86]
	Sonication (ultrasounds)	Rapture of hydrogen bonds in lignocellulose structure	[5,8,24,84,87,88]
	Spray drying with gamma radiation	Rapture of β-1,4 glycosidic bonds	[47,49,89,90]
	Pyrolysis	Carbonation of cellulose temperatures above 300 °C	[50,91]
Chemical	Acid hydrolysis	Decomposition of hemicellulose and dissolution of lignin	[34,35,37,50,84,91,92,93,94,95,96]
	Alkaline pretreatment	Saponification of lignocellulose, modification of lignin structure	[3,8,37,51,69,71,91,92,94,95,96,97,98,99,100,101,102,103,104,105,106,107]
	Oxidation and ozonation	Dissolution of lignin and hemicellulose, separation of cellulose crystals	[3,37,50,51,72,91,103,108,109,110]
	Treatment with ionic liquids	Separation of cellulose from lignocellulose	[3,6,70,71,72,73,74,111,112,113]
	Treatment with solvents (organic and others)	Rapture of hemicellulose bonds, dissolution of lignin	[3,34,37,48,51,71,75,76,77,78,79,80,100]
Physicochemical	Steam explosion	Dissolution of hemicellulose at 150 °C Dissolution of lignin at 180 °C and above	[3,4,37,47,78,114]
	Carbon dioxide explosion	Lignin and hemicellulose decomposition	[3,37,47,71,78,98,115]
	AFEX	Elimination of lignin and partially hemicellulose	[47,48,78,79,80,98,105,106,116]
Biological	White rot *Phanerochaete chrysosporium*, *Cyathus steercoreus*, *Pleurotus* spp.	Hemicellulose and lignin decomposition	[3,35,68,71,79,80,88,106,107,112,117,118,119,120,121,122,123,124,125,126,127,128,129,130,131,132,133,134,135,136]
	Brown rot *Gloeophyllum trabeum*	Lignin decomposition	
	Soft rot *Ascomycota*, *Deuteromycota*, *Trichoderma reesei*, *Chaetomium* sp., *Ceretocystis* sp.	Hemicellulose and lignin decomposition	
	Bacterial treatment	Hemicellulose and lignin decomposition	
	Enzymatic treatment	Hemicellulose and cellulose decomposition	[3,37,137,138,139,140,141]
	Pickling	Hemicellulose decomposition	

**Table 4:** Process parameters and efficiency of selected chemical methods of hydrolysis.

Hydrolysis Method/Applied Reagent	Feed Material	Process Parameters	Efficiency	Reference
1 stage: phosphoric acid 85% (100 mL) 2 stage: ethanol 96% (2 × 100 mL) 3 stage: neutralization with NaOH to pH = 5	Energetic willow (10 g dry matter)	1. 60 °C 45 min 2. centrifugation 3500 rpm	Feed material: about 1 g/L of glucose after 1 h and 5 g/L after 80 h Hydrolyzed material: about 4 g/L of glucose after 1 h and about 11 g/L after 70 h	[146]
NaOH 20 g dm^−3^ (100mL) Neutralization with HCl to pH = 5		12 h, ambient temp., centrifugation 3500 rpm	Feed material: about 1 g/L of glucose after 1 h and after 80 h Hydrolyzed material: about 2 g/L of glucose after 1 h and about. 5 g/L of glucose after 70 h	[146]
Ca(OH)_2_ 40 g dm^−3^ (100 mL) Neutralization with HCl to pH = 5		80 °C, 6 h	Feed material: about 1 g/L of glucose after 1 h and after 80 h Hydrolyzed material: about 2 g/L of glucose after 1 h and about 5 g/L of glucose after 70 h	[146]
H_2_SO_4_ 1% (100 mL)	Agave (100 g dry matter.)	200 °C, up to 20 h	1 g/L of glucose after 1 h; about 7 g/L of glucose after 10 h; then glucose concentration decreases	[147]
H_2_SO_4_ from 0.5–25% (100 mL)	Olive tree (100 g dry matter)	60–90 °C	An increase of reducing sugars concentration from about 2–30 g/100 g using 25% acid, maximum efficiency at 90 °C	[148]
H_3_PO_4_ from 2.5 to 10% (100 mL)	Potato peelings (100 g dry matter)	135–200 °C	Maximum efficiency: 35 g/L of glucose at 35 °C, Maximum efficiency: 45 g/L after 10 min, 10% acid	[149]
H_3_PO_4_ from 2–6% (100 mL)	Sugar cane (100 g dry matter)	122 °C, 6 h	2–3.5 g/L of glucose after 6 h (maximum efficiency when 6% acid is used)	[97]
H_2_SO_4_ from 5–13.5% (100 mL)	Model cellulose (100 dry matter)	180–240 °C	Hydrolysis results in an increase of glucose concentration by about 55–58% compared to untreated material	[50]
0.1 g Ca(OH)_2_/g dry matter	Rye straw	50–65 °C	Hydrolysis time 24 h results in an increase of glucose concentration by 4 times	[98]
0.1 g of Ca(OH)_2_/g dry matter,	Waste paper	150 °C	Conversion of lignin reaches 97% after 6 h of hydrolysis	[98]
24% KOH, 2% H_3_BO_3_	Corn straw	20 °C	Conversion of cellulose and hemicellulose reaches about 35% after 2 h of hydrolysis	[150]
H_2_SO_4_ 3% or NaOH 3%	Rice Waste	121 °C, 30 min	Reducing sugars concentration 49% (acid hydrolysis); 82% (acid hydrolysis)	[92]
H_2_SO_4_ 3% or NaOH 3%	Corn cobs	121 °C, 30 min	Reducing sugars concentration 45% (acid hydrolysis); 78% (acid hydrolysis)	[92]
H_2_SO_4_ 3% or NaOH 3%	Barley straw	121 °C, 30 min	Reducing sugars concentration 40% (acid hydrolysis); 65% (acid hydrolysis)	[92]
